# The Odds Exponential-Pareto IV Distribution: Regression Model and Application

**DOI:** 10.3390/e22050497

**Published:** 2020-04-25

**Authors:** Lamya A. Baharith, Kholod M. AL-Beladi, Hadeel S. Klakattawi

**Affiliations:** 1 Department of Statistics, Faculty of Science, King Abdulaziz University, Jeddah 21589, Saudi Arabia; kmmalbeladi@uj.edu.sa (K.M.A.-B.); hklakattawi@kau.edu.sa (H.S.K.); 2 Department of Statistics, Faculty Science, University of Jeddah, Jeddah 21959, Saudi Arabia

**Keywords:** Pareto IV, odds exponential-Pareto IV distribution, censored data, regression model, maximum likelihood, Jackknife method, residual analysis, global influence

## Abstract

This article introduces the odds exponential-Pareto IV distribution, which belongs to the odds family of distributions. We studied the statistical properties of this new distribution. The odds exponential-Pareto IV distribution provided decreasing, increasing, and upside-down hazard functions. We employed the maximum likelihood method to estimate the distribution parameters. The estimators performance was assessed by conducting simulation studies. A new log location-scale regression model based on the odds exponential-Pareto IV distribution was also introduced. Parameter estimates of the proposed model were obtained using both maximum likelihood and jackknife methods for right-censored data. Real data sets were analyzed under the odds exponential-Pareto IV distribution and log odds exponential-Pareto IV regression model to show their flexibility and potentiality.

## 1. Introduction

Pareto distribution was named after the Italian economist Vilfredo Pareto (1848–1923). The Pareto distribution has gained considerable attention in modeling many applications with heavy-tailed distributions, such as income distribution, earthquakes, forest fire areas, and disk drive sector errors [1,2]. The Pareto IV family is a general family of distributions. Pareto I, Pareto II, and Pareto III distributions are special cases of the Pareto IV family. Also, the Burr family can be regarded as a special case of Pareto IV (see, [3,4]). There are several studies in the literature generalizing the Pareto distribution to make it richer and more flexible for modeling data. These include the generalized Pareto [5], beta-Pareto [6], beta-generalized Pareto [7], Weibull–Pareto [8], gamma-Pareto [9,10], Kumaraswamy exponentiated Pareto [11], and exponentiated Weibull–Pareto distribution [12].

In recent works, adding new parameters to existing distributions or using different methods makes the resulting new distribution more appropriate and efficient for modeling the lifetime data. Many distributions have been generalized in the literature. These include the logit of the Kumaraswamy distribution [13], the generalized beta-generated distribution [14], the Weibull-G family of distribution [15], the gamma-exponentiated exponential distribution [16], and the transmuted Weibull-Pareto distribution [17]. Very recently, some new odd distributions were proposed in the literature, such as the odd Birnbaum–Saunders distribution [18], the odd Burr-III family of distributions [19], the odds exponential-log logistic distribution [20], the odd log-logistic-Fréchet distribution [21], the odd log-logistic-Burr XII distribution [22], the odd exponentiated half-logistic Burr XII distribution [23], the odd Lomax-G family of distributions [24], the odd Dagum-G family of distributions [25], and the odd log-logistic Lindley-exponential distribution [26].

This article used the transformed-transformer (T-X) family by Alzaatreh et al. [27] to introduce an odds exponential-Pareto IV distribution, in which the cumulative distribution function (CDF) is defined by

(1) $$G\left(x\right)=\int_{a}^{W(F(x))}r\left(t\right)\;dt=R\left\{W\left(F\left(x\right)\right)\right\},$$

where r(t) is the probability density function (PDF) of a random variable T∈[a,b], such that −∞≤a<b≤∞ and W(F(x)) is a function of any CDF, that takes different forms, see Alzaatreh et al. [27]. In this study, we consider the odds function form, $W\left(F\left(x\right)\right)=\frac{F(x)}{1-F(x)}$. That is, the CDF will be

(2) $$G\left(x\right)=\int_{0}^{\frac{F(x)}{1-F(x)}}r\left(t\right)\;dt=R\left\{\frac{F(x)}{1-F(x)}\right\},$$

and we considered the exponential distribution for $r\left(t\right)=\lambda e^{-\lambda t},\;t\geq 0,$ and $F\left(x\right)=1-{\left(1+{\frac{x}{\theta }}^{\frac{1}{a}}\right)}^{-\alpha },\;x>0,$ is the Pareto IV distribution with parameters (a,θ,α) in Equation (2). The resulting generated distribution will provide more flexibility in accommodating different types of the hazard function for the generated distribution. Also, this proposed distribution will be more suitable for modeling and fitting different real-life data

Therefore, we now define the odds exponential-Pareto IV (OEPIV) distribution with CDF given by

(3) $$G\left(x;\lambda ,a,\theta ,\alpha \right)=1-\exp\left\{-\lambda \left[{\left(1+{\left(\frac{x}{\theta }\right)}^{\frac{1}{a}}\right)}^{\alpha }-1\right]\right\},\;x>0\;.$$

The PDF of OEPIV is

(4) $$g\left(x;\lambda ,a,\theta ,\alpha \right)=\frac{\lambda \alpha }{a\theta }\exp\left(\lambda \right){\left(\frac{x}{\theta }\right)}^{\frac{1}{a}-1}{\left(1+{\left(\frac{x}{\theta }\right)}^{\frac{1}{a}}\right)}^{\alpha -1}\exp\left\{-\lambda {\left(1+{\left(\frac{x}{\theta }\right)}^{\frac{1}{a}}\right)}^{\alpha }\right\},\;x>0\;,$$

where λ>0, α>0 are the shape parameters, θ>0 is the scale parameter, and a>0 is the inequality parameter.

Recently, there has been a great deal of interest in the literature investigating the relationship between survival time and some other covariates, such as sex, weight, blood pressure, and many others. In a number of applications, different parametric regression models were used to estimate the effect of covariate variables on the survival time, including the log-location-scale regression model. The log-location-scale regression model is distinguished since it is commonly used in clinical trials and in many other fields of application. It is also widely used in engineering models where failure is accelerated by voltage, temperature, or other stress factors [28]. Several studies in the literature applied the log-location-scale regression model based on different distributions, such as the log-modified Weibull [29], the log-Weibull extended [30], the log-exponentiated Weibull [31], the log-Burr XII [32], the log-beta Weibull [33], the log-beta log-logistic [34], the log-Fréchet [35], the log-Exponentiated Fréchet [36], and the log-gamma-logistic [37]. Recent studies used the log-location-scale regression model built from the logarithm odd of the distribution. For instance, the odd log-logistic-Weibull [38], odd log-logistic generalized half normal [39], and odd Weibull [40].

This article is organized as follows: In Section 2, we define the survival and hazard functions of the OEPIV distribution with some graphical representations. We derived some of the OEPIV properties in Section 3. In Section 4, we explain the maximum likelihood estimation for parameters of the odds exponential-Pareto IV distribution. Simulation studies are provided to illustrate the performance of the OEPIV distribution in Section 5. In Section 6, we address the log odds exponential-Pareto IV (LOEPIV) distribution along with some of its statistical properties, in addition to introducing a log-location regression model based on LOEPIV and discussed its parameter estimates via maximum likelihood and Jackknife methods. In Section 7, three applications are analyzed to demonstrate the performance of the introduced new distribution and its regression model. Finally, we report our conclusions in Section 8.

## 2. The Odds Exponential-Pareto IV Distribution

The survival (SF) and hazard functions (HF) are, respectively, as follows:

(5) $$SF\left(x;\lambda ,a,\theta ,\alpha \right)=\exp\left\{-\lambda \left[{\left(1+{\left(\frac{x}{\theta }\right)}^{\frac{1}{a}}\right)}^{\alpha }-1\right]\right\},$$

(6) $$HF\left(x;\lambda ,a,\theta ,\alpha \right)=\frac{\lambda \alpha }{a\theta }{\left(\frac{x}{\theta }\right)}^{\frac{1}{a}-1}{\left(1+{\left(\frac{x}{\theta }\right)}^{\frac{1}{a}}\right)}^{\alpha -1}.$$

The Exponential-Pareto (EP) distribution [41] can be treated as a special case of OEPIV distribution by setting α=1 and 1/a=θ. For α=1, 1/a=σ and λ=1/β, we obtain the odds exponential-log logistic (OELL) distribution [20].

**Figure 1:**
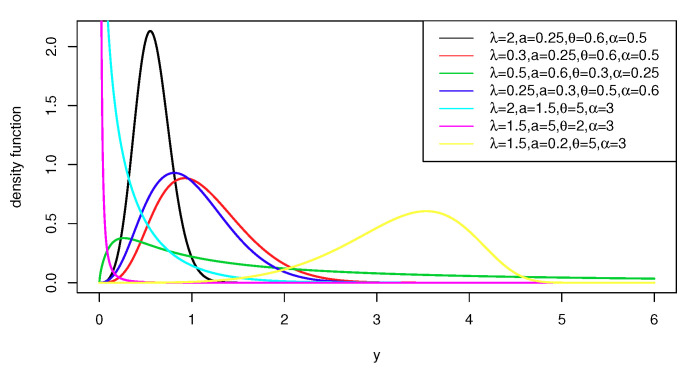
Density function plots of the OEPIV distribution.

**Figure 2:**
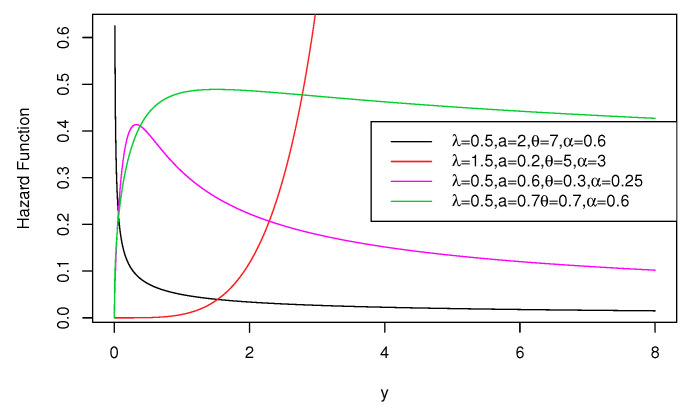
Hazard function plots of the OEPIV distribution.

Graphical representations of the PDF in Equation (4) and HF in Equation (6) are, respectively, shown in Figure 1 and Figure 2. From Figure 1, we note that the OEPIV distribution has different shapes at different parameter values, which indicate its great flexibility. Based on Figure 2, the OEPIV takes the following HF shapes: increasing, decreasing, and upside-down.

## 3. Statistical Properties

We discuss in this section some statistical properties of the OEPIV distribution.

### 3.1. The Quantile and Median

The quantile of the OEPIV distribution is computed as

(7) $$q^{OEPIV}=\theta {\left[{\left[\left(\frac{-\log(1-p)}{\lambda }\right)+1\right]}^{\frac{1}{\alpha }}-1\right]}^{a}.$$

Then, the median of the OEPIV distribution can be obtained by setting p=0.5 in Equation (7),

(8) $$Med=\theta {\left[{\left[\left(\frac{\log(2)}{\lambda }\right)+1\right]}^{\frac{1}{\alpha }}-1\right]}^{a}.$$

### 3.2. The Mode

The mode of the OEPIV distribution can be obtained by computing the derivative of the log PDF in Equation (4) with respect to x and equating to zero

$$\frac{d}{dx}\log g\left(x;\lambda ,a,\theta ,\alpha \right)=0$$

(9) $$\left(1/a-1\right)x+\frac{\left(\alpha -1\right){\left(x/\theta \right)}^{1/a-1}}{a\theta (1+{\left(x/\theta \right)}^{1/a})}-\frac{\lambda \alpha }{\theta a}{\left(x/\theta \right)}^{1/a-1}{\left(1+{\left(x/\theta \right)}^{1/a}\right)}^{\alpha -1}=0\;.$$

Thus, the mode can be obtained numerically by solving Equation (9).

### 3.3. The r-th Order Moment and Moment Generating Function

The *r*-th order raw moment is defined as

$${\mu^{\prime }}_{r}=\int_{0}^{\infty }x^{r}g\left(x;\lambda ,a,\theta ,\alpha \right)dx.$$

Thus,

$${\mu^{\prime }}_{r}=\int_{0}^{\infty }x^{r}\frac{\lambda \alpha }{a\theta }\exp\left(\lambda \right){\left(\frac{x}{\theta }\right)}^{\frac{1}{a}-1}{\left(1+{\left(\frac{x}{\theta }\right)}^{\frac{1}{a}}\right)}^{\alpha -1}\exp\left\{-\lambda {\left(1+{\left(\frac{x}{\theta }\right)}^{\frac{1}{a}}\right)}^{\alpha }\right\}dx.$$

Let

$u=\lambda {\left(1+{\left(\frac{x}{\theta }\right)}^{\frac{1}{a}}\right)}^{\alpha }\Rightarrow du=\frac{\lambda \alpha }{a\theta }{\left(\frac{x}{\theta }\right)}^{\frac{1}{a}-1}{\left(1+{\left(\frac{x}{\theta }\right)}^{\frac{1}{a}}\right)}^{\alpha -1}dx.$

Also, $x=\theta {\left[{\left(\frac{u}{\lambda }\right)}^{1/\alpha }-1\right]}^{a}.$

Thus, we put the above formulas in the integration to have

$${\mu^{\prime }}_{r}=e^{\lambda }\theta^{r}\int_{\lambda }^{\infty }{\left[{\left(\frac{u}{\lambda }\right)}^{1/\alpha }-1\right]}^{ar}e^{-u}du.$$

Using the binomial expansion of ${\left[{\left(\frac{u}{\lambda }\right)}^{1/\alpha }-1\right]}^{ar}$, we obtain

$${\mu^{\prime }}_{r}=\sum_{k=0}^{\infty }\left(\begin{array}{c} ar\\ k \end{array}\right){\left(-1\right)}^{k}e^{\lambda }\theta^{r}\lambda^{-\frac{ar-k}{\alpha }}\int_{\lambda }^{\infty }u^{(ar-k)/\alpha }e^{-u}du.$$

Using the gamma function definition,

$$\Gamma \left(s,x\right)=\int_{x}^{\infty }t^{s-1}e^{-t}dt.$$

Thus, the *r*-th moment can be written as

(10) $${\mu^{\prime }}_{r}=E\left(x^{r}\right)=\sum_{k=0}^{\infty }\left(\begin{array}{c} ar\\ k \end{array}\right){\left(-1\right)}^{k}e^{\lambda }\theta^{r}\lambda^{-\frac{ar-k}{\alpha }}\Gamma \left(\frac{ar-k}{\alpha }+1,\lambda \right).$$

Therefore, the moment generating function (mgf) can be obtained based on *r*-th moment of OEPIV distribution as

(11) $$M_{x}\left(t\right)=E\left(e^{tx}\right)=\sum_{r=0}^{\infty }\frac{t^{r}}{r!}{\mu^{\prime }}_{r}.$$

Substituting from Equation (10) into Equation (11), we find

$$M_{x}\left(t\right)=\sum_{r=0}^{\infty }\sum_{k=0}^{\infty }\left(\begin{array}{c} ar\\ k \end{array}\right){\left(-1\right)}^{k}\frac{{\left(\theta t\right)}^{r}}{r!}\lambda^{-\frac{ar-k}{\alpha }}e^{\lambda }\Gamma \left(\frac{ar-k}{\alpha }+1,\lambda \right)\;.$$

Then, the mean of the OEPIV distribution is

$${\mu^{\prime }}_{1}=E\left(x\right)=\sum_{k=0}^{\infty }\left(\begin{array}{c} a\\ k \end{array}\right){\left(-1\right)}^{k}e^{\lambda }\theta \lambda^{-\frac{a-k}{\alpha }}\Gamma \left(\frac{a-k}{\alpha }+1,\lambda \right)\;.$$

The mean, variance, skewness, and kurtosis of the OEPIV distribution for different values of λ, *a*, θ, and α are calculated in Table 1, to illustrate the effects on these measures.

### 3.4. Order Statistics

Suppose $X_{1},X_{2},X_{3},\ldots ,X_{n}$ is a random sample from the PDF in Equation (4). Let $X_{\left(1\right)},X_{\left(2\right)},X_{\left(3\right)},\ldots ,X_{\left(n\right)}$, denote the corresponding order statistic. The probability density function and the cumulative distribution function of the $k^{th}$ order statistic, say $Y=X_{\left(k\right)}$, given by

(12) $$f_{Y}\left(y\right)=\frac{n!}{(k-1)!(n-k)!}F^{k-1}\left(y\right){\left[1-F\left(y\right)\right]}^{n-k}f\left(y\right),$$

where f(y) and F(y) are the PDF and CDF of OEPIV distribution given by Equations (4) and (3), respectively. Using the binomial expansion of ${\left[1-F\left(y\right)\right]}^{n-k}$, given as follows

(13) $${\left[1-F\left(y\right)\right]}^{n-k}=\sum_{i=0}^{n-k}\left(\begin{array}{c} n-k\\ i \end{array}\right){\left(-1\right)}^{i}{\left[F\left(y\right)\right]}^{i}.$$

Substituting Equation (13) into (12), we have

(14) $$f_{Y}\left(y\right)=\frac{n!}{(k-1)!(n-k)!}f\left(y\right)\sum_{i=0}^{n-k}\left(\begin{array}{c} n-k\\ i \end{array}\right){\left(-1\right)}^{i}{\left[F\left(y\right)\right]}^{i+k-1}.$$

Substituting Equations (3) and (4) into (14), we obtain

(15) $$\begin{array}{c} f\left(y\right)=\frac{n!}{(k-1)!(n-k)!}\sum_{i=0}^{n-k}{\left(-1\right)}^{i}\left(\begin{array}{c} n-k\\ i \end{array}\right)\frac{\lambda \alpha }{a\theta }\exp\left(\lambda \right){\left(\frac{y}{\theta }\right)}^{\frac{1}{a}-1}{\left(1+{\left(\frac{y}{\theta }\right)}^{\frac{1}{a}}\right)}^{\alpha -1}\\ {\left[1-\exp\left\{-\lambda \left[{\left(1+{\left(\frac{y}{\theta }\right)}^{\frac{1}{a}}\right)}^{\alpha }-1\right]\right\}\right]}^{i+k-1}\exp\left\{-\lambda {\left(1+{\left(\frac{y}{\theta }\right)}^{\frac{1}{a}}\right)}^{\alpha }\right\} \end{array}$$

Using binomial expansion of ${\left[1-\exp\left\{-\lambda \left[{\left(1+{\left(\frac{y}{\theta }\right)}^{\frac{1}{a}}\right)}^{\alpha }-1\right]\right\}\right]}^{i+k-1}$, we get

$$\begin{array}{c} f\left(y\right)=\frac{n!}{(k-1)!(n-k)!}\sum_{j=0}^{\infty }\sum_{i=0}^{n-k}\left(\begin{array}{c} n-k\\ i \end{array}\right)\left(\begin{array}{c} i+k-1\\ j \end{array}\right){\left(-1\right)}^{i+j}\frac{\lambda \alpha }{a\theta }\exp\left(\lambda \right){\left(\frac{y}{\theta }\right)}^{\frac{1}{a}-1}{\left(1+{\left(\frac{y}{\theta }\right)}^{\frac{1}{a}}\right)}^{\alpha -1}\\ \exp\left\{-\lambda j\left[{\left(1+{\left(\frac{y}{\theta }\right)}^{\frac{1}{a}}\right)}^{\alpha }-1\right]\right\}\exp\left\{-\lambda {\left(1+{\left(\frac{y}{\theta }\right)}^{\frac{1}{a}}\right)}^{\alpha }\right\} \end{array}$$

(16) $$\begin{array}{c} f\left(y\right)=\frac{n!}{(k-1)!(n-k)!}\frac{\lambda \alpha }{a\theta }\sum_{j=0}^{\infty }\sum_{i=0}^{n-k}\left(\begin{array}{c} n-k\\ i \end{array}\right)\left(\begin{array}{c} i+k-1\\ j \end{array}\right){\left(-1\right)}^{i+j}\exp\left(\lambda \left(1+j\right)\right){\left(\frac{y}{\theta }\right)}^{\frac{1}{a}-1}\\ {\left(1+{\left(\frac{y}{\theta }\right)}^{\frac{1}{a}}\right)}^{\alpha -1}\exp\left\{-\lambda \left[{\left(1+{\left(\frac{y}{\theta }\right)}^{\frac{1}{a}}\right)}^{\alpha }\left(1+j\right)\right]\right\}. \end{array}$$

### 3.5. Rényi Entropy

The Rényi entropy of a random variable *X* represents a measure of variation of the uncertainty. It is given by

$$H_{R}\left(x\right)=\frac{1}{1-R}\log\left[\int_{0}^{\infty }g{\left(x\right)}^{R}dx\right],\;R>0,\;R\neq 1.$$

Using the PDF in Equation (4), we can write

$$g{\left(x\right)}^{R}={\left[\frac{\alpha \lambda \exp(\lambda )}{a\theta }\right]}^{R}{\left[{\left(\frac{x}{\theta }\right)}^{1/a-1}\right]}^{R}{\left[{\left(1+{\left(\frac{x}{\theta }\right)}^{1/a}\right)}^{\alpha -1}\right]}^{R}\exp\left\{-R\lambda {\left(1+{\left(\frac{x}{\theta }\right)}^{1/a}\right)}^{\alpha }\right\}.$$

$$I_{R}\left(x\right)=\int_{0}^{\infty }g{\left(x\right)}^{R}dx$$

$$=\int_{0}^{\infty }{\left[\frac{\alpha \lambda \exp(\lambda )}{a\theta }\right]}^{R}{\left[{\left(\frac{x}{\theta }\right)}^{1/a-1}\right]}^{R}{\left[{\left(1+{\left(\frac{x}{\theta }\right)}^{1/a}\right)}^{\alpha -1}\right]}^{R}\exp\left\{-R\lambda {\left(1+{\left(\frac{x}{\theta }\right)}^{1/a}\right)}^{\alpha }\right\}dx$$

Let $u=R\lambda {\left(1+{\left(\frac{x}{\theta }\right)}^{1/a}\right)}^{\alpha }$, so

$$I_{R}\left(x\right)=\frac{e^{\lambda R}}{R}{\left(\frac{\alpha \lambda }{a\theta }\right)}^{R-1}\int_{0}^{\infty }{\left(\frac{u}{R\lambda }\right)}^{R\left(1-\frac{1}{\alpha }\right)+\frac{1}{\alpha }-1}{\left[{\left(\frac{u}{R\lambda }\right)}^{\frac{1}{\alpha }}-1\right]}^{R(1-a)+a-1}e^{-u}du.$$

Using binomial expansion of ${\left[{\left(\frac{u}{R\lambda }\right)}^{\frac{1}{\alpha }}-1\right]}^{R(1-a)+a-1}$, given as follows

$${\left[{\left(\frac{u}{R\lambda }\right)}^{\frac{1}{\alpha }}-1\right]}^{R(1-a)+a-1}=\sum_{k=0}^{\infty }\left(\begin{array}{c} R(1-a)+a-1\\ k \end{array}\right){\left(-1\right)}^{k}{\left(\frac{u}{R\lambda }\right)}^{\frac{R(1-a)+a-1-k}{\alpha }}.$$

Thus, we put the above formula in the integration to have

$$I_{R}\left(x\right)=\frac{e^{\lambda R}}{R}{\left(\frac{\alpha \lambda }{a\theta }\right)}^{R-1}\sum_{k=0}^{\infty }\left(\begin{array}{c} R(1-a)+a-1\\ k \end{array}\right){\left(-1\right)}^{k}{\left(\frac{1}{R\lambda }\right)}^{\frac{1}{\alpha }\left(a\left(1-R\right)-k\right)+R-1}\int_{0}^{\infty }u^{\frac{1}{\alpha }\left(a\left(1-R\right)-k\right)+R-1}e^{-u}du$$

$$I_{R}\left(x\right)=e^{\lambda R}{\left(\frac{\alpha }{a\theta }\right)}^{R-1}\sum_{k=0}^{\infty }\left(\begin{array}{c} R(1-a)+a-1\\ k \end{array}\right)\frac{{\left(-1\right)}^{k}}{\lambda^{1/\alpha (a(1-R)-k)}}\frac{\Gamma (1/\alpha (a(1-R)-k)+R)}{R^{1/\alpha ((1-R)-k)+R}}.$$

$$\log\left(I_{R}\left(x\right)\right)=\lambda R+\left(R-1\right)\log\left(\frac{\alpha }{a\theta }\right)+\log\left[\sum_{k=0}^{\infty }\left(\begin{array}{c} R(1-a)+a-1\\ k \end{array}\right)\frac{{\left(-1\right)}^{k}}{\lambda^{1/\alpha (a(1-R)-k)}}\frac{\Gamma (1/\alpha (a(1-R)-k)+R)}{R^{1/\alpha ((1-R)-k)+R}}\right].$$

The Rényi entropy of the OEPIV distribution is

$$H_{R}\left(x\right)=\frac{\lambda R}{1-R}-\log\left(\frac{\alpha }{a\theta }\right)+\frac{1}{1-R}\log\left[\sum_{k=0}^{\infty }\left(\begin{array}{c} R(1-a)+a-1\\ k \end{array}\right)\frac{{\left(-1\right)}^{k}}{\lambda^{1/\alpha (a(1-R)-k)}}\frac{\Gamma (1/\alpha (a(1-R)-k)+R)}{R^{1/\alpha ((1-R)-k)+R}}\right].$$

## 4. Estimation of the OEPIV Parameters

We assume that $x_{1},x_{2},\ldots ,x_{n}$ is a random sample from the OEPIV distribution. Then, the log-likelihood (*ℓ*) for ϕ=(λ,a,θ,α) is

(17) $$\ell =n\log\left(\lambda \right)+n\log\left(\alpha \right)-n\log\left(a\right)-n\log\left(\theta \right)+n\lambda +\left(\frac{1}{a}-1\right)\sum_{i=1}^{n}\log\left(\frac{x_{i}}{\theta }\right)+\left(\alpha -1\right)\sum_{i=1}^{n}\log\left(h_{i}\right)-\lambda \sum_{i=1}^{n}{\left(h_{i}\right)}^{\alpha },$$

where $h_{i}=1+{\left(\frac{x_{i}}{\theta }\right)}^{1/a}$. The likelihood equations are given by

(18) $$\frac{\partial \ell }{\partial \lambda }=\frac{n}{\lambda }+n-\sum_{i=1}^{n}{\left(h_{i}\right)}^{\alpha },$$

(19) $$\frac{\partial \ell }{\partial a}=-\frac{n}{a}-\frac{1}{a^{2}}\sum_{i=1}^{n}\log\left(\frac{x_{i}}{\theta }\right)-\frac{\left(\alpha -1\right)}{a^{2}}\sum_{i=1}^{n}\frac{1}{h_{i}}{\left(\frac{x_{i}}{\theta }\right)}^{1/a}\ln\left(\frac{x_{i}}{\theta }\right)+\frac{\lambda \alpha }{a^{2}}\sum_{i=1}^{n}h_{i}^{\alpha -1}(\frac{x_{i}}{\theta }{)}^{1/a}\ln\left(\frac{x_{i}}{\theta }\right),$$

(20) $$\frac{\partial \ell }{\partial \theta }=-\frac{n}{\theta }-\frac{(1/a)-1}{\theta }-\frac{\left(\alpha -1\right)}{a\theta }\sum_{i=1}^{n}\frac{1}{h_{i}}{\left(\frac{x_{i}}{\theta }\right)}^{1/a}+\frac{\lambda \alpha }{a\theta }\sum_{i=1}^{n}{\left(\frac{x_{i}}{\theta }\right)}^{\left(1/a\right)}h_{i}^{\alpha -1},$$

and

(21) $$\frac{\partial \ell }{\partial \alpha }=\frac{n}{\alpha }+\sum_{i=1}^{n}\log\left(h_{i}\right)-\lambda \sum_{i=1}^{n}h_{i}^{\alpha }\log\left(h_{i}\right).$$

We can obtain maximum likelihood (ML) estimates of the parameters by directly maximizing Equation (17) using the nlm or optim functions in R package or by solving Equations (18)–(21). Under standard regularity conditions, we can obtain approximate intervals estimation of the parameters using multivariate normal distribution $N_{4}\left(0,J{\left(\hat{\phi }\right)}^{-1}\right)$ by numerically evaluating the elements of the 4×4 observed information matrix J(ϕ) at $\hat{\phi }$, $J\left(\phi \right)=\left(-\frac{\partial^{2}\ell }{\partial \phi_{j}\partial \phi_{k}}\right)$. In addition, the likelihood ratio (LR) test can be applied to discriminate between nested models.

## 5. Simulation Studies

We conducted a Monte Carlo simulation to illustrate the performance of the ML parameter estimates of the OEPIV distribution. That is, we randomly generated 10,000 samples with size 30, 50, 100, 200, and 500 from the OEPIV distribution for two different sets of parameter values as follows:

SetI:λ=0.3,a=0.4,θ=0.5,α=0.2.

SetII:λ=0.2,a=0.1,θ=0.6,α=0.15.

The estimates for the parameters were obtained along with their calculated bias and mean square error (MSE), given by

$${\hat{Bias}}_{b}=\frac{1}{n}\sum_{i=1}^{n}\left({\hat{b}}_{i}-b\right),$$

$${\hat{MSE}}_{b}=\frac{1}{n}\sum_{i=1}^{n}{\left({\hat{b}}_{i}-b\right)}^{2},$$

where b=λ,θ,a,α. The results of the simulation are displayed in Table 2. We concluded from these results that the empirical means tend to the true value of the parameters as the sample size increases. In addition, the MSEs and biases decreased as we increased the sample size.

## 6. The Log Odds Exponential-Pareto IV Regression Model

If X is a random variable from the OEPIV distribution, as given in Equation (4), then Y=log(X) is a random variable that has a LOEPIV distribution with the transformation parameter σ=a and μ=log(θ). Therefore, the PDF and CDF of the LOEPIV distribution are as follows:

(22) $$f\left(y;\lambda ,\alpha ,\sigma ,\mu \right)=\frac{\lambda \alpha }{\sigma }\exp\left(\lambda \right)\exp\left(\frac{y-\mu }{\sigma }\right){\left(1+\exp\left(\frac{y-\mu }{\sigma }\right)\right)}^{\alpha -1}\exp\left\{-\lambda {\left(1+\exp\left(\frac{y-\mu }{\sigma }\right)\right)}^{\alpha }\right\},$$

(23) $$F\left(y;\lambda ,\alpha ,\sigma ,\mu \right)=1-\exp\left(\lambda \right)\exp\left\{-\lambda {\left(1+\exp\left(\frac{y-\mu }{\sigma }\right)\right)}^{\alpha }\right\},\;-\infty <y<\infty$$

where σ>0 is the scale parameter, λ>0, α>0 are the shape parameters, and −∞<μ<∞ is the location parameter. The LOEPIV model becomes the log exponential-Pareto (LEP) distribution for α=1. The PDF (for −∞<y<∞) of the LEP distribution with parameters λ>0, σ>0 and −∞<μ<∞, is

$$f\left(y\right)=\frac{\lambda }{\sigma }\exp\left(\lambda \right)\exp\left(\frac{y-\mu }{\sigma }\right)\exp\left\{-\lambda \left(1+\exp\left(\frac{y-\mu }{\sigma }\right)\right)\right\}$$

The SF and HF are given by

(24) $$SF\left(y;\lambda ,\alpha ,\sigma ,\mu \right)=\exp\left(\lambda \right)\exp\left\{-\lambda {\left(1+\exp\left(\frac{y-\mu }{\sigma }\right)\right)}^{\alpha }\right\},$$

(25) $$HF\left(y;\lambda ,\alpha ,\sigma ,\mu \right)=\frac{\lambda \alpha }{\sigma }\exp\left(\frac{y-\mu }{\sigma }\right){\left(1+\exp\left(\frac{y-\mu }{\sigma }\right)\right)}^{\alpha -1}.$$

The following are the properties for the LOEPIV distribution:

The quantile of the LOEPIV distribution

(26) $$y=\sigma \ln\left[{\left(1-\frac{1}{\lambda }\ln\left(1-p\right)\right)}^{\frac{1}{\alpha }}-1\right]+\mu \;.$$

The mode of the LOEPIV distribution

(27) $$\frac{d}{dy}\log f\left(y;\sigma ,\mu \right)=\frac{1}{\sigma }\left[1+\left(\alpha -1\right)\frac{\exp\left(\frac{y-\mu }{\sigma }\right)}{1+\exp\left(\frac{y-\mu }{\sigma }\right)}-\lambda \alpha {\left(1+\exp\left(\frac{y-\mu }{\sigma }\right)\right)}^{\alpha -1}\exp\left(\frac{y-\mu }{\sigma }\right)\right]=0\;.$$

Then, the mode can be obtained by solving Equation (27) numerically.

The median of the LOEPIV distribution

(28) $$Med=\sigma \ln\left[{\left(1+\frac{1}{\lambda }\ln\left(2\right)\right)}^{\frac{1}{\alpha }}-1\right]+\mu \;.$$

The mgf of LOEPIV distribution

$$M_{Y}\left(t\right)=\int_{-\infty }^{\infty }\exp(ty)f(y;\lambda ,\alpha ,\sigma ,\mu )dy.$$

Thus,

$$=\int_{-\infty }^{\infty }\exp\left(ty\right)\frac{\lambda \alpha }{\sigma }\exp\left(\lambda \right)\exp\left(\frac{y-\mu }{\sigma }\right){\left(1+\exp\left(\frac{y-\mu }{\sigma }\right)\right)}^{\alpha -1}\exp\left\{-\lambda {\left(1+\exp\left(\frac{y-\mu }{\sigma }\right)\right)}^{\alpha }\right\}dy.$$

Substituting $u={\left(1+\exp\left(\frac{y-\mu }{\sigma }\right)\right)}^{\alpha }$$\Rightarrow du=\frac{\alpha }{\sigma }\exp\left(\frac{y-\mu }{\sigma }\right){\left(1+\exp\left(\frac{y-\mu }{\sigma }\right)\right)}^{\alpha -1},$ will reduce the above integration to

$$M_{Y}\left(t\right)=\lambda e^{\lambda }\exp\left(t\mu \right)\int_{1}^{\infty }{\left(u^{1/\alpha }-1\right)}^{t\sigma }e^{-\lambda u}du.$$

Then, using the binomial expansion

$${\left(u^{1/\alpha }-1\right)}^{t\sigma }=\sum_{j=0}^{\infty }\left(\begin{array}{c} t\sigma \\ j \end{array}\right){\left(-1\right)}^{j}{\left(u^{1/\alpha }\right)}^{t\sigma -j},$$

$M_{Y}\left(t\right)$ can be rewritten as

$$M_{Y}\left(t\right)=\lambda e^{\lambda }\exp\left(t\mu \right)\sum_{j=0}^{\infty }\left(\begin{array}{c} t\sigma \\ j \end{array}\right){\left(-1\right)}^{j}\int_{1}^{\infty }{\left(u^{1/\alpha }\right)}^{t\sigma -j}e^{-\lambda u}du.$$

Using the gamma function. Thus, the mgf of LOEPIV distribution is as follows

$$M_{Y}\left(t\right)=e^{\lambda }\exp\left(t\mu \right)\sum_{j=0}^{\infty }\left(\begin{array}{c} t\sigma \\ j \end{array}\right){\left(-1\right)}^{j}{\left(\frac{1}{\lambda }\right)}^{\frac{t\sigma -j}{\alpha }}\Gamma \left(\left(\frac{t\sigma -j}{\alpha }\right)+1,\lambda \right).$$

The standardized random variable for y in Equation (22) is defined as z=(y−μ)/σ, then z has the following PDF

(29) $$f\left(z\right)=\lambda \alpha \exp\left(\lambda \right)\exp\left(z\right){\left(1+\exp\left(z\right)\right)}^{\alpha -1}\exp\left\{-\lambda {\left(1+\exp\left(z\right)\right)}^{\alpha }\right\},\;-\infty <z<\infty$$

with SF given as

(30) $$SF\left(z\right)=\exp\left(\lambda \right)\exp\{-\lambda {\left(1+\exp\left(z\right)\right)}^{\alpha }\}.$$

Hence, a linear location-scale regression model with response variable $y_{i}$ and explanatory vector $x_{i}={\left(x_{i1},\ldots ,x_{ip}\right)}^{T}$ can be defined as

(31) $$y_{i}=\beta^{T}x_{i}+\sigma z_{i},i=1,2,\ldots ,n,$$

where $z_{i}$ is the random error with PDF in Equation (24), $\beta ={\left(\beta_{1},\ldots ,\beta_{p}\right)}^{T}$, and σ>0, λ>0, and α>0 are the unknown parameters. $y_{i}$ is the location of $\mu_{i}=\beta^{T}x_{i}$ and the location vector $\mu ={\left(\mu_{1},\ldots ,\mu_{n}\right)}^{T}$ can be represented as a linear model $\mu =\beta^{T}x$, in which ${\left(x_{1},\ldots ,x_{n}\right)}^{T}$ is the known model matrix. Therefore, the SF of $Y_{i}|x$ is expressed as:

$$SF\left(y_{i}|x\right)=\exp\left(\lambda \right)\exp\left\{-\lambda {\left(1+\exp\left(\frac{y_{i}-\beta^{T}x_{i}}{\sigma }\right)\right)}^{\alpha }\right\}.$$

### 6.1. Estimation of the LOEPIV Regression Model

#### 6.1.1. ML Method

For the right-censored lifetime data, we have $t_{i}=\min\left(f_{i},c_{i}\right)$, where $f_{i}$ is the lifetime and $c_{i}$ is the censoring time, then, we have $y_{i}=\log\left(t_{i}\right)$ for the ith individual i=1,…,n. If we have a random sample with n observations $\left(y_{1},\tau_{1},x_{1}\right)$,...,$\left(y_{n},\tau_{n},x_{n}\right)$, where $\tau_{i}=\left\{\begin{array}{ccc} 1 & \mathrm{for} & y_{i}=\log\left(t_{i}\right)\\ 0 & \mathrm{for} & y_{i}=\log\left(c_{i}\right) \end{array}\right.$, and assuming the censoring and lifetimes are independent and random. Then, the likelihood function for the regression model in (31) with $\theta ={\left(\lambda ,\alpha ,\sigma ,\beta \right)}^{T}$ assuming right censoring is as follows:

$$L\left(\theta \right)=\prod_{i=1}^{n}{\left(f\left(y_{i}\right)\right)}^{\tau_{i}}{\left(SF\left(y_{i}\right)\right)}^{1-\tau_{i}},$$

where $f(y_{i})$ and $SF(y_{i})$ are given by Equations (17) and (19) of $Y_{i}$, respectively. The *ℓ* for θ reduces to

(32) $$\begin{array}{c} \ell =r\log\left(\lambda \right)+r\log\left(\alpha \right)-r\log\left(\sigma \right)+r\lambda +\sum_{i=1}^{n}\tau_{i}\left[z_{i}+\left(\alpha -1\right)\log\left(1+\exp\left(z_{i}\right)\right)-\lambda {\left(1+\exp\left(z_{i}\right)\right)}^{\alpha }\right]\\ +\sum_{i=1}^{n}\left(1-\tau_{i}\right)\log\left(\exp\left(\lambda \right)\exp\left[-\lambda {\left(1+\exp\left(z_{i}\right)\right)}^{\alpha }\right]\right), \end{array}$$

where $\sum_{i=1}^{n}\tau_{i}=r$ represents the uncensored data, and $z_{i}=\left(y_{i}-\beta^{T}x_{i}\right)/\sigma$. The ML estimate for the parameter vector θ could be obtained using an optimization algorithm that maximizes Equation (32).

#### 6.1.2. Jackknife Method

The jackknife technique was developed by Quenouille (1949) to estimate the bias of an estimator. It is an alternative method to estimate the LOEPIV parameters based on “leaving one out”.

Suppose that $\hat{\theta }$ is the parameter estimation of the whole sample and ${\hat{\theta }}_{-i}$ is the parameter estimation when we dropped the ith observation from the data. That is, the pseudo-value of the ith observation is obtained as

(33) $${\tilde{\theta }}_{i}=n\hat{\theta }-\left(n-1\right){\hat{\theta }}_{-i}.$$

Then, the jackknife estimate of θ is the mean of pseudo-values, denoted ${\hat{\theta }}^{*}$ is

(34) $${\hat{\theta }}^{*}=\frac{1}{n}\sum_{i=1}^{n}{\tilde{\theta }}_{i}.$$

For more details, see [42,43,44].

### 6.2. Sensitivity Analysis: Global Influence

Global influence, introduced by [45], is used to conduct a sensitivity analysis that represents the diagnostic effect depending on the case deletion. Case deletion measures the impact of dropping the ith observation from the data set on the estimate of the parameters. That is, this method is based on comparing the difference of $\hat{\theta }$ and ${\hat{\theta }}_{-i}$ where ${\hat{\theta }}_{-i}$ is the estimated parameters when the ith observation is dropped from data. If ${\hat{\theta }}_{-i}$ is distant from $\hat{\theta }$, then this case is considered as influential. The case deletion model for the LOEPIV regression Model (31) is

(35) $$Y_{J}=\beta^{T}x_{i}+\sigma Z_{i};J=1,2,\ldots ,n,J\neq i.$$

We denote the ML estimate of θ when the ith observation is dropped by ${\hat{\theta }}_{-i}={\left({\hat{\lambda }}_{\left(i\right)},{\hat{\alpha }}_{\left(i\right)},{\hat{\sigma }}_{\left(i\right)},{\hat{\beta }}_{\left(i\right)}\right)}^{T}$. Then, we describe two methods of global influence below.

#### 6.2.1. Generalized Cook Distance

Generalized Cook distance (GD) is the first measure of global influence and is defined as

$$GD_{i}\left(\theta \right)={\left(\left({\hat{\theta }}_{-i}-\hat{\theta }\right)\right)}^{T}\left\{\ddot{M}\left(\hat{\theta }\right)\right\}\left({\hat{\theta }}_{-i}-\hat{\theta }\right),$$

where $\ddot{M}\left(\hat{\theta }\right)$ denotes the observed information matrix.

#### 6.2.2. Likelihood Distance

Likelihood distance (LD) measures the differences between $\hat{\theta }$ and ${\hat{\theta }}_{-i}$, and is given by

$$LD_{i}\left(\theta \right)=2\left\{\ell \left(\hat{\theta }\right)-\ell \left({\hat{\theta }}_{-i}\right)\right\},$$

where $\ell ({\hat{\theta }}_{-i})$ is the log likelihood function of θ when the ith observation is dropped from the data.

### 6.3. Residual Analysis

In the regression model, checking the assumptions and appropriateness of the fitted model is an essential step. Therefore, we used residual analysis to check the assumptions and detect outlier observations. In this study, we consider the following types.

#### 6.3.1. Martingale Residual

Barlow and Prentice [46] proposed the martingale residual as

$$r_{M_{i}}=\delta_{i}+\log\left(SF\left(y_{i};\hat{\theta }\right)\right),$$

where $\delta_{i}$ denotes the censor indicator, where $\delta_{i}=0$, if the ith observation is censored, and $\delta_{i}=1$, if the ith observation is not censored, and $SF(y_{i};\hat{\theta })$ denotes the SF for the regression model. Therefore, the martingale residual of the LOEPIV regression model is

(36) $$r_{M_{i}}=\left\{\begin{array}{ccc} 1+\log[\exp\left(\lambda \right)\exp\left(-\lambda {\left(1+\exp\left({\hat{z}}_{i}\right)\right)}^{\alpha }\right)] & \mathrm{if} & i\in l\mathrm{if}etime\\ \log[\exp\left(\lambda \right)\exp\left(-\lambda {\left(1+\exp\left({\hat{z}}_{i}\right)\right)}^{\alpha }\right)] & \mathrm{if} & i\in censored \end{array}\right.$$

where $r_{M_{i}}$ has a range between −∞ and 1 and has skewness. Thus, the transformation of $r_{M_{i}}$ will be used to reduce the skewness.

#### 6.3.2. Deviance Residual

This is a further improvement of the martingale residual, which reduces the skewness and make it more symmetrical, around zero. It can be expressed as

$$r_{D_{i}}=sign\left(r_{M_{i}}\right)\sqrt{-2[r_{M_{i}}+\delta_{i}\log\left(\delta_{i}-r_{M_{i}}\right)]},$$

where $r_{M_{i}}$ is defined in Equation (36), and the deviance for the LOEPIV regression model is

$$r_{D_{i}}=\left\{\begin{array}{ccc} sign(1+\log\left[\exp\left(\lambda \right)\exp\left(-\lambda {\left(1+\exp\left({\hat{z}}_{i}\right)\right)}^{\alpha }\right)\right])\\ {\left\{\begin{array}{c} -2\{1+\log\left[\exp\left(\lambda \right)\exp\left(-\lambda {\left(1+\exp\left({\hat{z}}_{i}\right)\right)}^{\alpha }\right)\right]\\ +\log(-\log\left[\exp\left(\lambda \right)\exp\left(-\lambda {\left(1+\exp\left({\hat{z}}_{i}\right)\right)}^{\alpha }\right)\right])\} \end{array}\right\}}^{\frac{1}{2}} & \mathrm{if} & i\in \;l\mathrm{if}etime\\ sign(\log\left[\exp\left(\lambda \right)\exp\left(-\lambda {\left(1+\exp\left({\hat{z}}_{i}\right)\right)}^{\alpha }\right)\right])\\ {\left\{-2\left\{\log\left[\exp\left(\lambda \right)\exp\left(-\lambda {\left(1+\exp\left({\hat{z}}_{i}\right)\right)}^{\alpha }\right)\right]\right\}\right\}}^{\frac{1}{2}} & \mathrm{if} & i\in censored. \end{array}\right.$$

## 7. Simulation Study for the Log Odds Exponential-Pareto IV Regression Model

**Figure 3:**
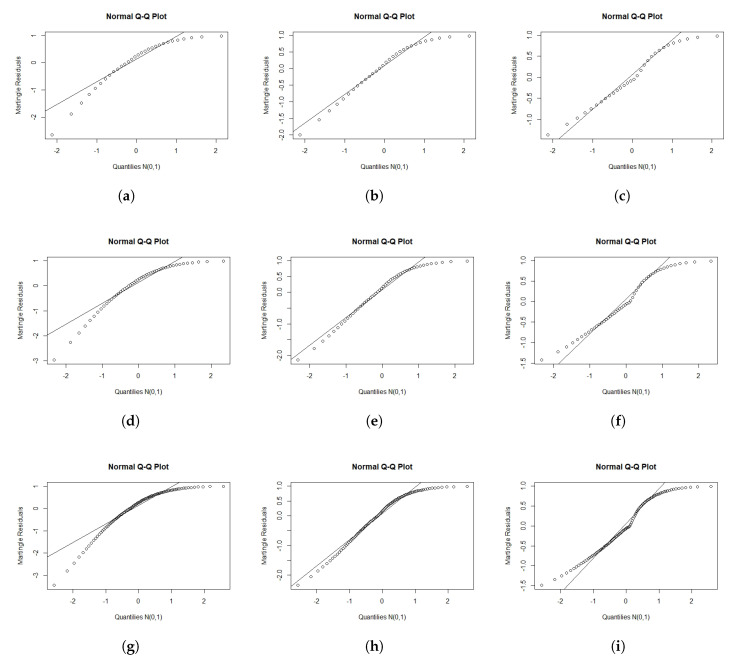
Normal probability plots (NPP) for $r_{M_{i}}$ for different sample sizes (n) and censoring levels (c). (**a**) n = 30; c = 0.1 (**b**) n = 30; c = 0.3 (**c**) n = 30; c = 0.5 (**d**) n = 50; c = 0.1 (**e**) n = 50; c = 0.3 (**f**) n = 50; c = 0.5 (**g**) n = 100; c = 0.1 (**h**) n = 100; c = 0.3 (**i**) n = 100; c = 0.5.

**Figure 4:**
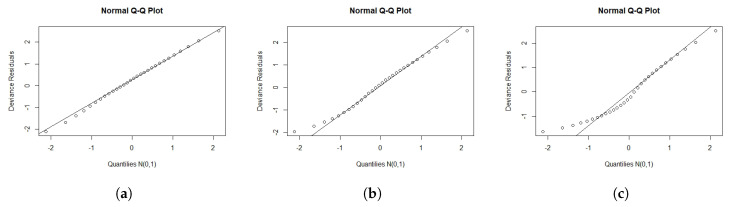

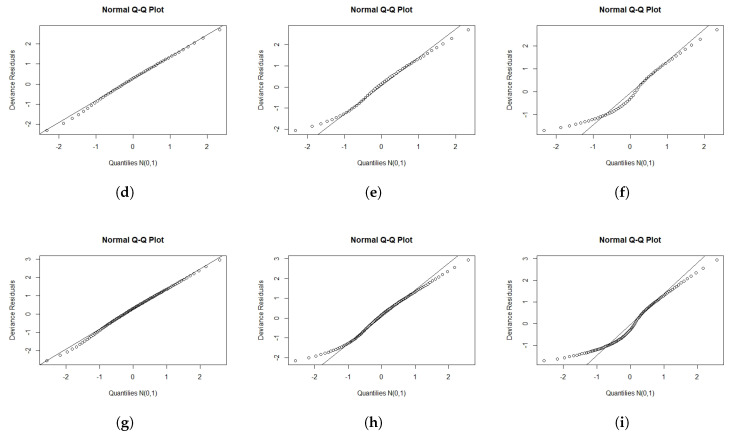
NPP for $r_{D_{i}}$ for different sample sizes (n) and censoring levels (c). (**a**) n = 30; c = 0.1 (**b**) n = 30; c = 0.3 (**c**) n = 30; c = 0.5 (**d**) n = 50; c = 0.1 (**e**) n = 50; c = 0.3 (**f**) n = 50; c = 0.5 (**g**) n = 100; c = 0.1 (**h**) n = 100; c = 0.3 (**i**) n = 100; c = 0.5.

We performed a Monte Carlo simulation to explore the empirical distribution of the $r_{M_{i}}$ and $r_{D_{i}}$ for different values of n and different censoring levels. The lifetimes $t_{1},\ldots ,t_{n}$ were from the OEPIV distribution in Equation (4), and $x_{i}$ was generated from uniform (0,1). We sampled the censoring times $c_{1},\ldots ,c_{n}$ from uniform (0,ρ), where ρ was adjusted until we obtained the required censoring level. For each fit, the log lifetimes were obtained as $y_{i}=\min\left\{\log\left(t_{i}\right),\log\left(c_{i}\right)\right\}$. We generated 1000 samples. For each selection of $n,\lambda ,\alpha ,\sigma ,\beta_{0}$, and $\beta_{1}$, and the censoring levels. The simulation was conducted for n=30, 50, and 100 with λ=0.3, α=0.36, σ=0.6, $\beta_{0}=-0.6$, and $\beta_{1}=1$, and the censoring levels 0.1, 0.3, and 0.5. Figure 3 and Figure 4 present normal probability plots (NPP) for the residuals. These figures show that the $r_{D_{i}}$ empirical distribution provided more agreement with the standard normal distribution (SND) compared to $r_{M_{i}}$. $r_{D_{i}}$ also approached the SND as we increased the sample size or decreased the censoring level.

## 8. Applications

We analyzed three real data sets to investigate the flexibility of the OEPIV distribution and the LOEPIV regression model.

### 8.1. The Strength of Glass Fibers Data

This data was analyzed by [47], and it represents the strength of glass fibers with the length 1.5 cm. This data consists of 63 observations.

We will compare the fits of the OEPIV with the Pareto IV, Weibull BurrXII (WBXII) in [48], Weibull Frechet (WFr) in [49], Weibull Lomax (WL) in [50], Odd exponential-weibull (OE-W), Odd exponential-normal (OE-N) in [51], and Gamma distributions.

We considered the following criteria to compare these distributions: the values of the negative log-likelihood function ($-\hat{\ell }$), Akaike information criterion (AIC), and corrected Akaike Information Criterion (CAIC). The smaller the values for these statistics, the better the fit to the data.

**Figure 5:**
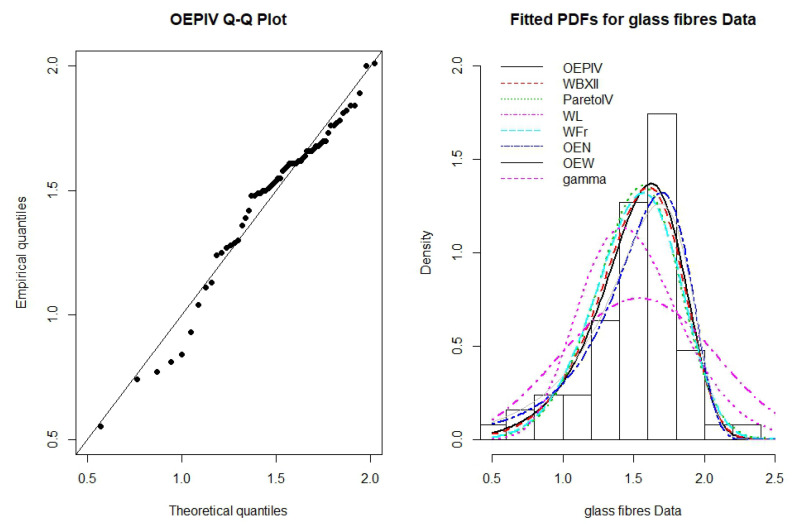
QQ-plot of the OEPIV model and the estimated PDFs of the OEPIV and other competitive distributions for the glass fibers data.

The ML estimates, standard errors (SE), $-\hat{\ell }$, AIC and CAIC statistics for the OEPIV, WBXII, WL, WFr, Pareto IV,OE-W, OE-N, and Gamma distributions are reported in Table 3. From the results in Table 3, it is clear that the OEPIV distribution provides better fit for the data having lowest AIC and CAIC values and could be selected as a more appropriate model than other models. Figure 5 displays the QQ-plot of the OEPIV distribution and the estimated PDFs of the fitted distributions. It is clear from these plots that the OEPIV captures the skewness of the glass fibers data than other competitive fitted distributions.

### 8.2. Sum of Skin Folds Data

The authors of [52] discussed this data set, and it represents 102 male and 100 female athletes collected at the Australian Institute of Sports, provided by Richard Telford and Ross Cunningham.

**Figure 6:**
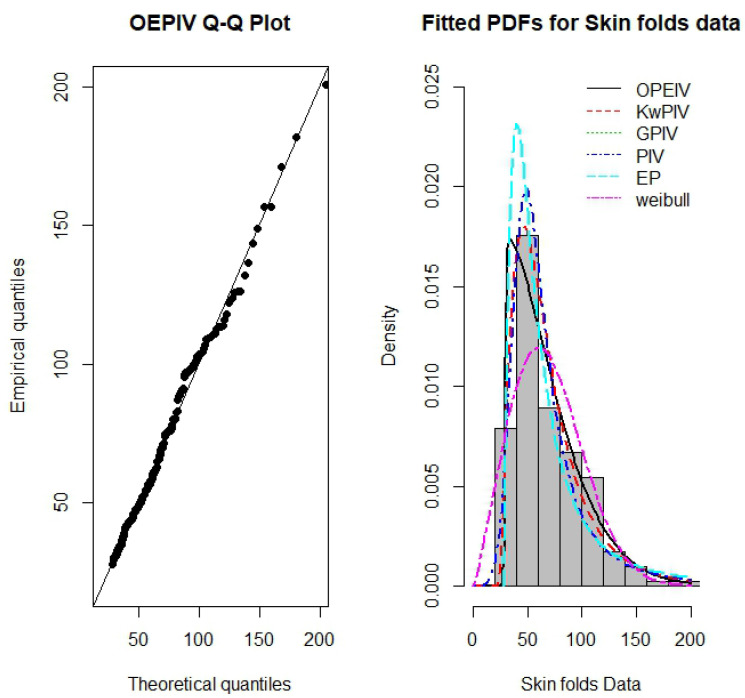
QQ-plot of the OEPIV distribution and the estimated PDFs of the OEPIV and other competitive distributions for the skin folds data.

We compare the ML estimates and their corresponding SE, and the values of the ($-\hat{\ell }$), and the AIC and CAIC statistic for fitted OEPIV distribution with the results of the Kumaraswamy Pareto-IV (KwPIV) in [53], gamma-Pareto IV (GPIV) [10], Pareto IV (PIV) in [53], and exponentiated Pareto (EP) distributions provided in [54], and the Weibull distribution. These results are reported in Table 4. From the results in Table 4, it is clear that the OEPIV distribution provides the lowest AIC and CAIC values among those of the fitted distributions. Therefore, OEPIV could be selected as the best modal for this data. Figure 6 displays the QQ-plot of the OEPIV distribution and the estimated PDFs of the fitted distributions. It is clear from these plots that the OEPIV provides a good fit to this data.

### 8.3. Stanford Heart Transplant Data

This data was obtained from Kalbfleisch and Prentice [55] and has information on n = 103 patients. The patient’s survival time was specified as the number of days from the acceptance into a heart transplant program to death. The following are associated with each patient: $y_{i}$: log survival time (days); $status_{i}$: censoring indicator (1 = dead, 0 = censoring); $x_{i1}$: is the age (in years); $x_{i2}$: is the prior surgery coded as (0 = No, 1 = Yes); and $x_{i3}$: is the transplant coded as (0 = No, 1 = Yes). This data set was used by [38], [35], and [36] for illustrating the log-odd log-logistic Weibull (LOLLW), log-Fréchet (LF), and log-exponentiated Fréchet (LEF) regression models. The LOEPIV regression model will be compared with the log-Weibull (LW), LEP, LOLLW, LF, and LEF regression models.

That is, we present the results from fitting the following model

$$y_{i}=\beta_{0}+\beta_{1}x_{i1}+\beta_{2}x_{i2}+\beta_{3}x_{i3}+\sigma z_{i},$$

where $y_{i}$ follows the LOEPIV distribution in Equation (22).

**Figure 7:**
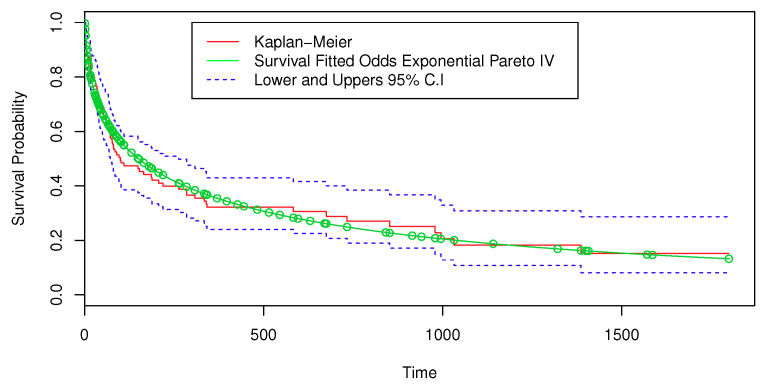
Estimated SF based on the OEPIV distribution and the Kaplan–Meier (KM) model for the heart transplant data.

To examine the suitability of the proposed model, a plot of the empirical SF estimates from the Kaplan–Meier (KM) model and the SF from the fitted OEPIV model are displayed in Figure 7. Therefore, we concluded that the logarithm of times to event follow the LOEPIV distribution.

#### 8.3.1. ML and Jackknife Estimation

The estimates, their corresponding SE, *p*-values, AIC, CAIC, and Bayesian Information Criterion (BIC) statistics for the LOEPIV, LEF, LOLLW, LF, LW and LEP regression models are shown in Table 5. The results demonstrated that the LOEPIV regression model had the lowest AIC, CAIC, and BIC. This shows the superiority of the LOEPIV model over other models. The LR test can be used to discriminate between LOEPIV and LEP regression models since they are nested.That is, the LR statistic for testing the hypotheses $H_{0}:\alpha =1$ versus $H_{1}:H_{0}$ is not true given in Table 6 and rejects the LEP model in favor of the LOEPIV model.

Table 7 lists the jackknife parameter estimates of the LOEPIV model, their corresponding SE and 95% confidence intervals. Based on the results in Table 5 and Table 7, we observed that the explanatory variables $x_{1}$, $x_{2}$, and $x_{3}$ are significant for the fitted model and both methods displayed similar estimates.

**Figure 8:**
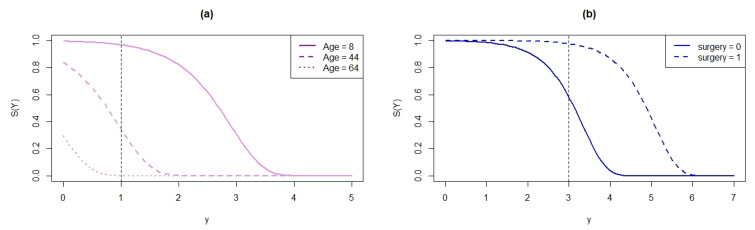

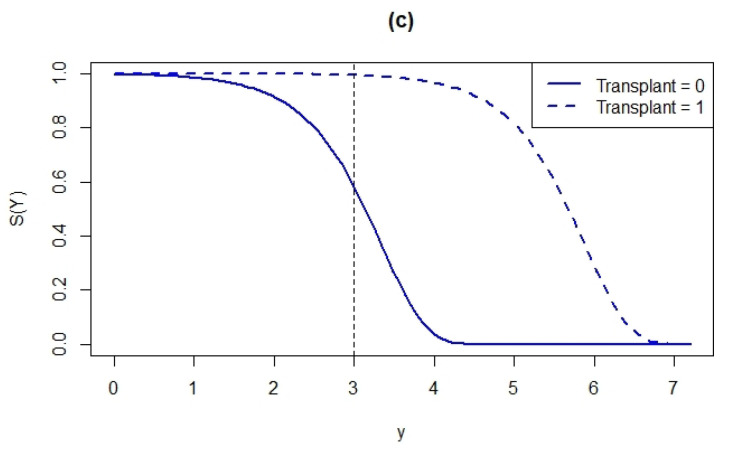
Fitted SF from the LOEPIV regression model (**a**) for $x_{1}$ = age, (**b**) for $x_{2}$ = surgery, (**c**) for $x_{3}$ = transplant.

The plots of the SF that corresponded to the explanatory variables for the fitted LOEPIV regression model are presented in Figure 8. From Figure 8a, we observed that $\hat{S}\left(1|age\;=\;8\right)\;=\;0.96808$, which means that ≈ 97% of the patients who are 8 years old will be thriving when y = 1 (≈3 days). However, for patients between 44 and 64 years old, $\hat{S}\left(1|age\;=\;44\right)=\;0.34676$ and $\hat{S}\left(1|age\;=\;64\right)=\;0.00064$, which indicated that the percentages of living patients at y = 1 decreased to 34% and 0.06%, respectively. These results indicate decreases in survival of the patients as their age increased. Similarly, Figure 8b,c indicated that approximately 58% of patients who did not have surgery or receive a transplant were thriving at y = 3 (≈21 days). Furthermore, for the patients who undertook surgery, we observed that approximately 98% of them were thriving at y = 3, while patients that received a transplant, $\hat{S}\left(3|transplant\;=\;1\right)=\;0.9943$, increased to 99% at y = 3 in the survival percentage. Therefore, it can be stated that receiving a heart transplant increased the survival time when undergoing surgery.

#### 8.3.2. Global Influence Analysis

**Figure 9:**
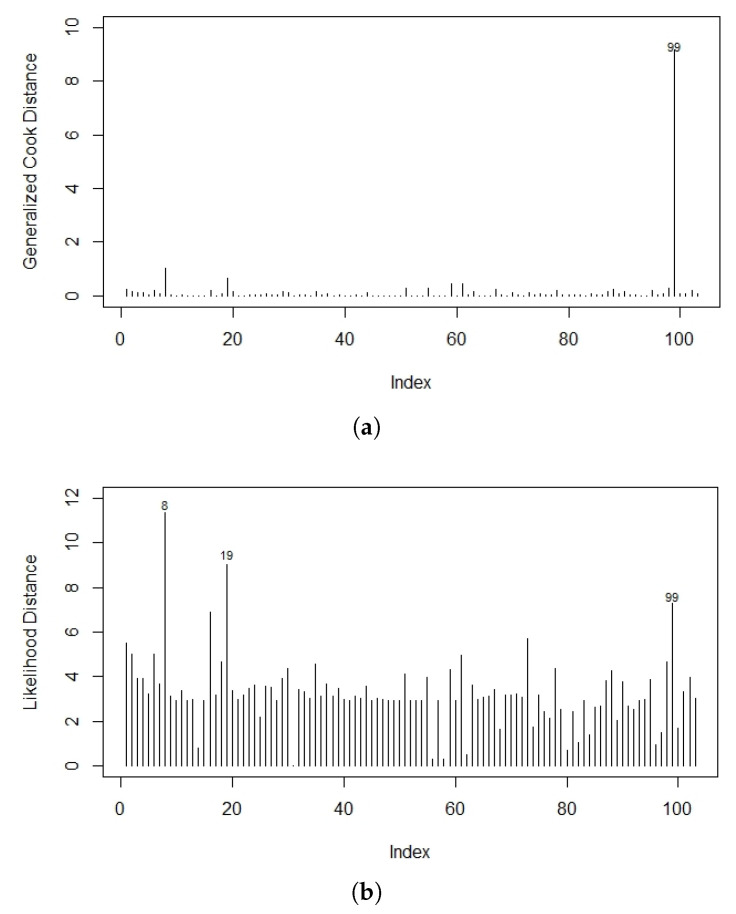
The index plot of (**a**) $GD_{i}\left(\theta \right)$ and (**b**) $LD_{i}\left(\theta \right)$ for the LOEPIV regression model.

The case deletion measures $GD_{i}\left(\theta \right)$ and $LD_{i}\left(\theta \right)$ were numerically computed and Figure 9 represents the influence measure index plots. It is clear that case 99 could be an influential observation in the LOEPIV regression model.

#### 8.3.3. Residual Analysis

**Figure 10:**
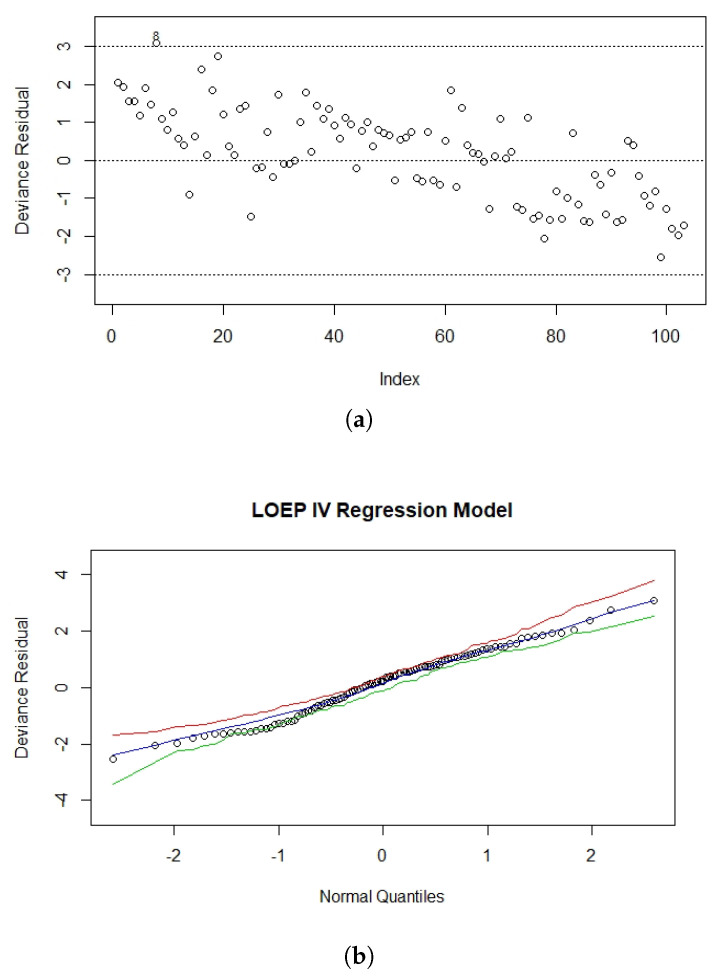
The index plot of (**a**) the deviance residual and (**b**) the NPP for the deviance residual with envelopes.

In order to detect possible outlaying observations, a plot for the $r_{D_{i}}$ versus the observations index is shown in Figure 10a. This demonstrated that almost all of the observations fall within (−3, 3), except for observation 8. Therefore, observation 8 was a possible outlier. Figure 10b shows the NPP for the deviance residuals with a generated envelope. Approximately all of the observations fell inside the envelope, which indicated that the proposed model was appropriate to fit the heart transplant data.

## 9. Concluding Remarks

In this article, we introduced the odd exponential-Pareto IV distribution. We derived some of its statistical and mathematical properties. The model parameters were estimated using the ML method, and simulation studies were carried out to examine the performance of the ML estimators based on biases and mean squared errors. Moreover, a new log-location regression model for censored data based on the OEPIV distribution was introduced. The ML and jackknife estimation methods for right censored data were used to estimate the unknown parameters of the new regression model. The model assumptions were checked using martingale and deviance residuals. Furthermore, generalized Cook and likelihood distance measures were defined to detect the influence observations for the regression model. Finally, we analyzed three real data sets to examine the usefulness of the OEPIV distribution and LOEPIV regression model. The results demonstrated that the OEPIV distribution outperformed other competitive distributions in terms of goodness of fit. In addition, the LOEPIV regression model provides a good fit for the Stanford heart transplant data.

## Figures and Tables

**Table 1:** Mean, variance, skewness, and kurtosis of OEPIV model selected parameter values.

λ	*a*	θ	α	Mean	Variance	Skewness	Kurtosis
2	2.5	0.5	1.5	1.1281	23.5677	0.5281	0.0424
2	3.5	0.5	1.5	4.3493	192.0261	0.1223	0.0665
2	4.5	0.5	1.5	24.8511	488.3011	13.3934	7.6745
2	2.5	2.5	1.5	5.6405	589.1917	0.5281	0.0424
2	2.5	3.5	1.5	7.8967	1154.8158	0.5281	0.0424
2	2.5	0.5	1.5	1.1281	23.5677	0.5281	0.0424
0.5	2.5	1.5	1.5	1.1153	5.3631	0.7241	0.4752
0.5	2.5	1.5	2.5	0.9486	9.6007	0.0298	0.0131
0.5	2.5	1.5	4.5	0.8567	13.2012	0.0302	0.0084
1.5	3.5	0.5	1.5	3.0317	47.0037	0.5800	0.3771
2.5	3.5	0.5	1.5	7.7388	568.5549	0.1148	0.0424
3.5	3.5	0.5	1.5	42.8019	1795.2542	4.7337	2.5407

**Table 2:** Parameter estimates, along with their MSE, and bias for two different cases with different sample sizes.

		Set I			Set II		
		**Estimate**	**MSE**	**Bias**	**Estimate**	**MSE**	**Bias**
n=30	λ	0.7646	34.3149	0.4646	0.4444	1.1410	0.2444
	*a*	0.1806	0.1159	−0.2194	0.0347	0.0086	−0.0653
	θ	1.0773	1009.5916	0.5773	0.6595	0.0570	0.0595
	α	0.0778	0.0364	−0.1222	0.0440	0.0374	−0.1060
n=50	λ	0.5774	1.1837	0.2774	0.3526	0.4563	0.1526
	*a*	0.2333	0.0893	−0.1667	0.0495	0.0074	−0.0505
	θ	0.6825	0.7605	0.1825	0.6366	0.0235	0.0366
	α	0.1008	0.0228	−0.0992	0.0631	0.0161	−0.0869
n=100	λ	0.4324	0.3672	0.1324	0.2628	0.0909	0.0628
	*a*	0.3072	0.0540	−0.0928	0.0683	0.0051	−0.0317
	θ	0.6042	0.2970	0.1042	0.6147	0.0132	0.0147
	α	0.1430	0.0128	−0.0570	0.0953	0.0105	−0.0547
n=200	λ	0.3535	0.0982	0.0535	0.2243	0.0221	0.0243
	*a*	0.3532	0.0256	−0.0468	0.0830	0.0028	−0.0170
	θ	0.5463	0.1018	0.0463	0.6054	0.0064	0.0054
	α	0.1718	0.0057	−0.0282	0.1211	0.0058	−0.0289
n=500	λ	0.3156	0.0140	0.0156	0.2069	0.0038	0.0069
	*a*	0.3847	0.0082	−0.0153	0.0942	0.0010	−0.0058
	θ	0.5149	0.0211	0.0149	0.6015	0.0020	0.0015
	α	0.1911	0.0017	−0.0089	0.1403	0.0020	−0.0097

**Table 3:** Maximum likelihood (ML) estimates, SE in (), $-\hat{\ell }$, and Akaike information criterion (AIC) and corrected Akaike Information Criterion (CAIC) statistics for the glass fibers data.

Distribution		ML Estimate and SE in ()			$-\hat{\ell }$	AIC	CAIC
OEPIV	λ = 0.0401	*a* = 0.2862	θ = 1.1455	α = 2.1549	13.9507	35.902	36.591
	(0.0810)	(0.1368)	(0.4016)	((1.4014)			
WBXII	*a* = 0.0026	*b* = 1.8888	α = 1.6077	β = 2.7409	14.3035	36.607	37.297
	(0.0032)	(0.7680)	(0.3760)	(1.0100)			
WL	*a* = 581.4052	*b* = 5.1752	α = 17.5336	β = 110.7104	14.934	37.868	38.558
	(28.2900)	(0.2010)	(102.1130)	(659.3920)			
WFr	*a* = 1.4762	*b* = 16.8561	α = 0.3865	β = 0.2436	15.5005	39.001	39.691
	(4.7820)	(20.4850)	(0.7990)	(0.2850)			
Pareto IV	*a* = 0.1626	θ = 2.3513	α = 10.2153	-	15.4781	36.956	37.363
	(0.0187)	(0.4477)	(9.9080)				
OE-W	λ = 0.0721	β = 1.9603	-	-	16.4613	36.922	37.123
	(0.0162)	(0.0940)					
OE-N	λ = 0.0121	σ = 0.7385	-	-	17.5979	39.195	39.396
	(0.0043)	(0.0364)					
Gamma	β = 17.4411	θ = 11.5748	-	-	23.9515	51.9031	52.1031
	(3.0783)	(2.0725)					

**Table 4:** ML estimates, SE in (), $-\hat{\ell }$, and AIC and CAIC statistics for skin folds data.

Distribution		ML Estimate and SE in ()				$-\hat{\ell }$	AIC	CAIC
OEPIV	λ = 0.348	*a* = 0.024	θ = 29.579	α = 0.036	-	944.2687	1896.537	1896.740
	(0.090)	(0.006)	(0.678)	(0.010)			
KwPIV	*a* = 2.928	*b* = 21.746	α = 0.023	γ = 0.060	θ = 23.430	945.200	1900.401	1900.707
	(1.188)	(33.283)	(0.019)	(0.033)	(4.633)		
GPIV	*c* = 0.520	α = 81.355	σ = 0.098	-	-	950.007	1906.014	1906.135
	(0.198)	(8.071)	(0.035)				
PIV	α = 0.463	γ = 0.182	θ = 46.812	-	-	956.333	1918.666	1918.787
	(0.183)	(0.041)	(5.595)				
EP	*c* = 28	α = 2.155	θ = 2.737	-	-	951.878	1907.757	1907.878
		(0.154)	(0.298)				
Weibull	α = 2.2635	θ = 78.2664	-	-	-	975.2427	1954.485	1954.545
	(0.1159)	(2.5832)						

**Table 5:** The ML estimates, SE in (), *p*-values in [], AIC, CAIC, and ayesian Information Criterion (BIC) statistics of the log odds exponential-Pareto IV (LOEPIV), log-exponentiated Fréchet (LEF), log-odd log-logistic Weibull (LOLLW), log-Fréchet (LF), log-Weibull (LW), and log exponential-Pareto (LEP) regression models for the heart transplant data.

Models	λ	α	σ	$\beta_{0}$	$\beta_{1}$	$\beta_{2}$	$\beta_{3}$	AIC	CAIC	BIC
	1.3754	0.1257	0.5569	3.5186	−0.0539	1.7494	2.5405	343.42	344.61	361.87
LOEPIV	(1.9087)	(0.0974)	(0.1689)	(1.0747)	(0.0192)	(0.5524)	(0.3621)			
				[0.00106]	[0.00507]	[0.00154]	[<0.001]			
	-	6.2746	3.5882	8.6744	−0.0624	0.8910	2.7241	346.72	347.59	362.53
LEF	-	(7.5737)	(1.4492)	(3.5491)	(0.0206)	(0.5059)	(0.3780)			
	-	-	-	[0.016]	[0.002]	[0.078]	[<0.001]			
	-	4.62831	6.20325	8.74485	−0.07692	1.40550	2.59196	347.59	348.47	363.40
LOLLW	-	(3.5307)	(4.6851)	(1.7603)	(0.0199)	(0.5745)	(0.3884)			
	-	-	-	[<0.001]	[<-0.001]	[0.016]	[<0.001]			
	-	-	1.7457	4.2129	−0.0431	0.6902	2.6572	349.15	349.77	362.33
LF	-	-	(0.1484)	(0.9153)	(0.0189)	(0.5034)	(0.3782)			
	-	-	-	[<0.001]	[0.023]	[0.170]	[<0.001]			
	-	-	1.4658	7.9742	−0.0924	1.2143	2.5375	353.42	354.03	366.59
LW	-	-	(0.13148)	(0.93397)	(0.02061)	(0.64700)	(0.37336)			
	-	-	-	[<0.001]	[<0.001]	[0.063]	[<0.001]			
	0.1439	-	1.4655	5.1321	−0.0923	1.214127	2.537713	355.42	356.29	371.22
LEP	(1.1088)	-	(0.1314)	(11.3276)	(0.0206)	(0.6469)	(0.3733)			
	-	-	-	[0.6505]	[<0.001]	[0.061]	[<0.001]			

**Table 6:** LR statistic for heart transplant.

Heart Transplant	Hypotheses		Statistic w	*p*-Values
LOEPIV vs. LEP	$H_{0}:\alpha =1$ versus $H_{1}:H_{0}$ is not true		13.9922	0.00018

**Table 7:** The Jackknife parameter estimates of the LOEPIV regression model.

Parameter	Estimate	SE	95% Confidence Intervals
λ	1.4043	1.5262	(0.0000, 4.3957)
α	0.0838	0.0988	(0.0000, 0.2775)
σ	0.6586	0.1885	(0.2891, 1.0281)
$\beta_{0}$	3.8616	1.1072	(1.6915, 6.031)
$\beta_{1}$	-0.0536	0.0196	(−0.0921, −0.0152)
$\beta_{2}$	1.7304	0.5262	(0.6989, 2.7619)
$\beta_{3}$	2.5563	0.3881	(1.7955, 3.3172)
